# Supplementary material for: A Nitrogen-Rich DOPO-Based Derivate for Increasing Fire Resistance of Epoxy Resin with Comparable Transparency
Source: Materials (Basel). 2023 Jan 5;16(2):519. doi: 10.3390/ma16020519 (PMC9865245; doi:10.3390/ma16020519)
Supplement: Supplementary file 1 [file materials-16-00519-s001.zip › materials-2073273-supplementary.pdf]

Supporting information

A nitrogen-rich DOPO-based derivate for reducing the fire hazard of epoxy resin with comparable transparency

Jiayi Lu, Boyu Cai, Wendi Xu, Luze Wang, Zhonglin Luo, Biaobing Wang*

Jiangsu Key Laboratory of Environmentally Friendly Polymeric Materials, School of Materials Science and Engineering, Jiangsu Collaborative Innovation Center of Photovoltaic Science and Engineering, Changzhou University, Changzhou, Jiangsu, 213164, People's Republic of China.

*Corresponding author: Biaobing Wang

Tel./Fax: +86 0519-8633-0075

E-mail: biaobing@cczu.edu.cn (B.W.)

Materials

Bisphenol-A epoxy resin (DGEBA, epoxy value of 0.51 mol/100 g) was purchased from Hangzhou Wuhuigang Adhesive Co., Ltd. (Hangzhou China). 2-Furaldehyde (FA, AR), 5-amino-1H-tetrazol (ATZ, AR), 9,10-dihydro-9-oxa-10-phosphaphenanthrene 10-oxide (DOPO, 97%) and 4,4'-Dia-minodiphenyl methane (DDM,99%) as the curing reagent were obtained from Aladdin reagent Co., Ltd. (Shanghai, China). Anhydrous ethanol was obtained from Sinopharm Chemical Reagent Co., Ltd. (Shanghai, China). All chemicals were used as received without further purification.

Physical characterizations

Differential scanning calorimetry (DSC) was carried out on a Perkin-Elmer DSC 4000 (PE, USA) to analyze the non-isothermal curing kinetics of epoxy composites under nitrogen atmosphere, the mass of all samples was about 5 mg. Furthermore, Different Scanning Calorimerter (DSC) was employed to investigate T_g of the pure epoxy resin and flame retardant EP blends. Samples need to be removed from thermal history.The samples were heated from 30 to 200 °C with a heating rate of 10 °C/min. The mass of all the samples was ca. 10 mg.

The vertical burning (UL-94) testing was measured according to GB/T 2048-2008 on a CZF-3 instrument (Shine Ray Instrument Co. Ltd., Nanjing, China) with the sample dimension of 130 ×13 × 3 mm^3. The burning time was the average value of at least five replicates.

The limited oxygen Index (LOI) testing was carried out according to GB/T 2046.1-2008 on a HC-2 oxygen index meter (Jiang Ning Co. Ltd., Nanjing, China) with a size of 130×6.5×3 mm^3, and the LOI values were the average value of at least five replicates.

The combustion behavior of the samples (about 40 g) with a size of 10×10×3 mm^3 were analyzed on a FTT cone calorimeter (Fire Testing Technology, Britain) according to the ISO 5660-1 standard at an external heat flux of 35 kW/m^2. The average value of

three parallel tests was collected as the testing result.

The non-isothermal curing process was performed on a Perkin-Elmer TGA 4000 (Waltham, Massachusetts, USA) in the temperature range of 30-250 °C with different heating rate of 5, 10, 15 and 20 °C/min.

The thermogravimetry-infrared spectroscopy (TG-IR) was achieved by the combined system of TGA 4000 thermogravimetric analyser and the Spectrum Ⅱ FTIR spectrophotometer, specimen (about 10 mg) was heated from 30 to 700 °C at a heating rate of 10 °C/min under a nitrogen atmosphere.

Scanning electron microscopy (SEM) was performed to observe the surface morphology of char residues on the SUPRA55 scanning electron microscope. All sample surfaces were sprayed platinum twice.

Dynamic mechanical analysis (DMA) was carried out on the Perkin Elmer DMA 8000 (PE, USA). The instrument needed to be calibrated once before use. Three point bending mode was used for sample measurement. The constant frequency was 1HZ and the amplitude was 20 μm.The prepressure was set to 0.02 N. The samples were heated from 30~250 °C at a heating rate of 10 °C/min.

X-ray photoelectron spectroscopy (XPS) was employed to characterize the chemical composition of the samples on a ESCALAB 250Xi system (Thermo Fischer Scientific, US) with a Al Kα excitation radiation (hν = 1486.6 eV).

The tensile testing was performed on a GT-TCS-2000 tensile testing machine (High Speed Railway Testing Instrument Co., Ltd., Dongguang, China) with an extension rate of 2 mm/min, ten specimens with thickness of 3 mm was tested. Flexural measurement was carried out using a three-point bending mode on a CMT6104 universal testing machine (MTS Systems Co. Ltd., China) at bending speed of 2mm/min, and the dimensions of sample was 127 × 12.7 × 3 mm^3. The data of tensile and flexural properties were achieved from the average value of at least five replicates.

The transmittance of the samples (thickness of 3 mm) was recorded on the UV1800 UV-Vis scanning spectrophotometer (SHIMADZU, Japan) in the range of 200~800 nm.

1. Fourier transform infrared (FTIR)

Fourier transform infrared (FTIR) spectra were collected on a Perkin Elmer instrument (Waltham, MA, USA) using KBr pellets, and the samples was scanned 32 times in a wavenumber range from 4000 to 400 cm^{-1} with a resolute of 4 cm^{-1}.

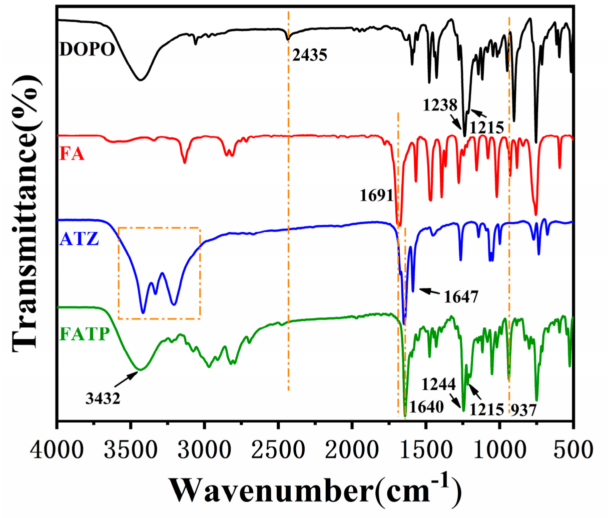

Figure S1 FTIR spectra of FA, ATZ, DOPO and FATP

2. ^{1}H and ^{31}P nuclear magnetic resonance

The ^{1}H and ^{31}P Nuclear magnetic resonance (NMR) spectra were recorded on a 400 MHz superconducting Fourier NMR spectrometer (Bruker AVANCE II, Germany), with DMSO-d6 as deuterated reagent.

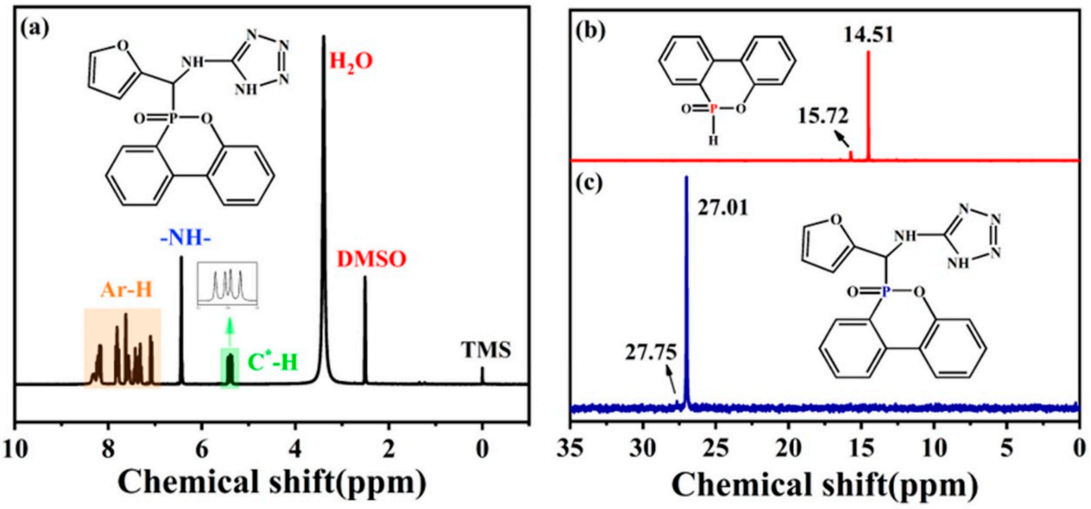

Figure S2. ^{1}H NMR spectrum of FATP (a), ^{31}P NMR spectra of DOPO (b) and FATP(c)

3. TGA and DTG curves under nitrogen atmosphere

The thermal stability was evaluated on a Perkin-Elmer TGA 4000 (Waltham, Massachusetts, USA) under nitrogen atmosphere, with temperature range of 30-700 °C and a heating rate of 10 °C/min.

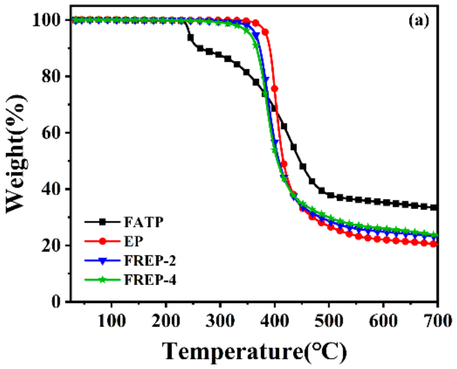

Figure S3 TGA and DTG curves under nitrogen atmosphere

4. Raman Spectra

Laser Raman spectroscopy (LRs) was obtained through DXR2xi Laser Raman spectrometer (Thermo Fischer Scientific, US) at the range of 500-3000 cm^{-1} with a excitation wavelength of 532 nm.

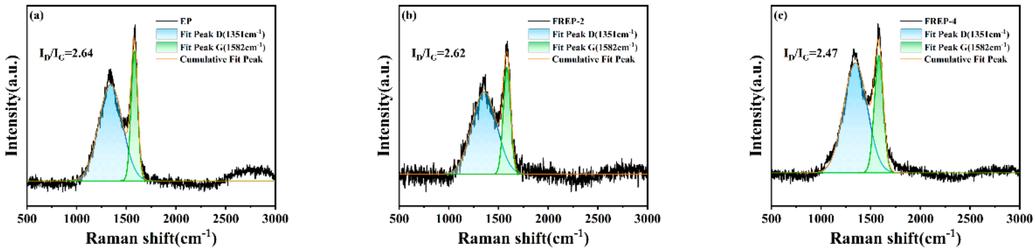

Figure S4 Raman spectra of the char residue for pure EP and FREPs

5. X-ray photoelectron spectroscopy (XPS)

X-ray photoelectron spectroscopy (XPS) was employed to characterize the chemical composition of the samples on a ESCALAB 250Xi system (Thermo Fischer Scientific, US) with an Al Kα excitation radiation (hν = 1486.6 eV).

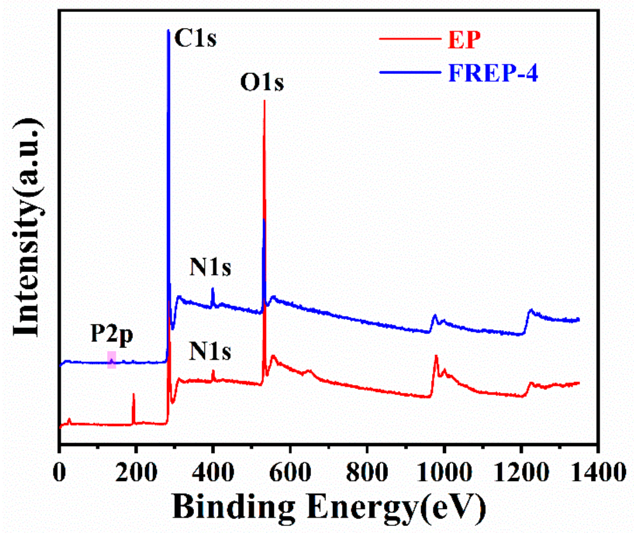

Figure S5. XPS spectra of the char residue

6. C1s, N1s, O1s, and P2p spectra

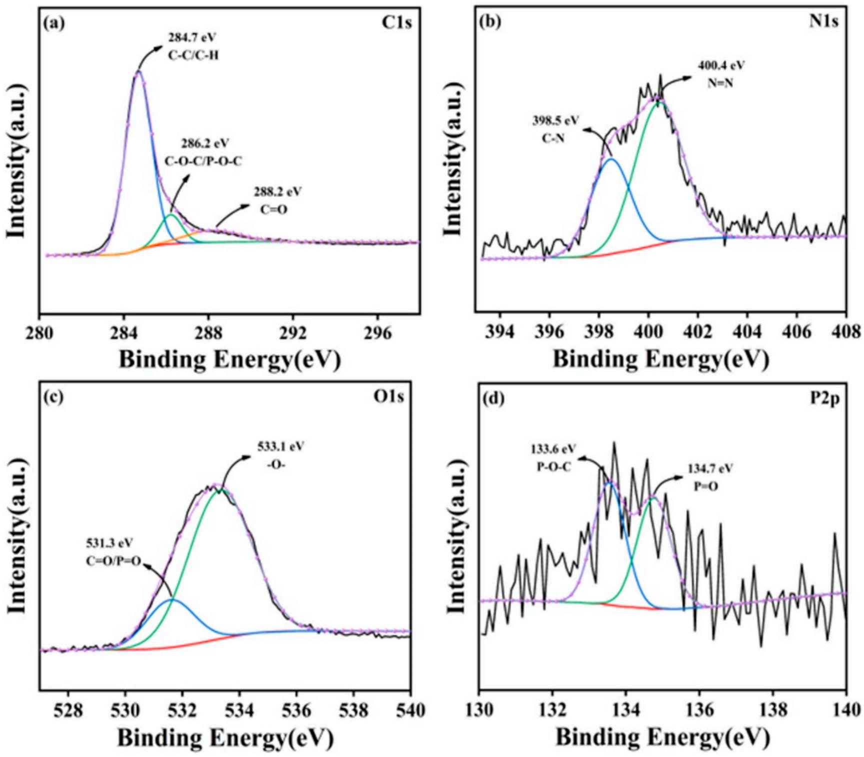

Figure S6 C1s(a), N1s(b), O1s (c), and P2p (d) spectra of char residues of FREP-4

7. Peak intensity of some typical flammable volatiles with time

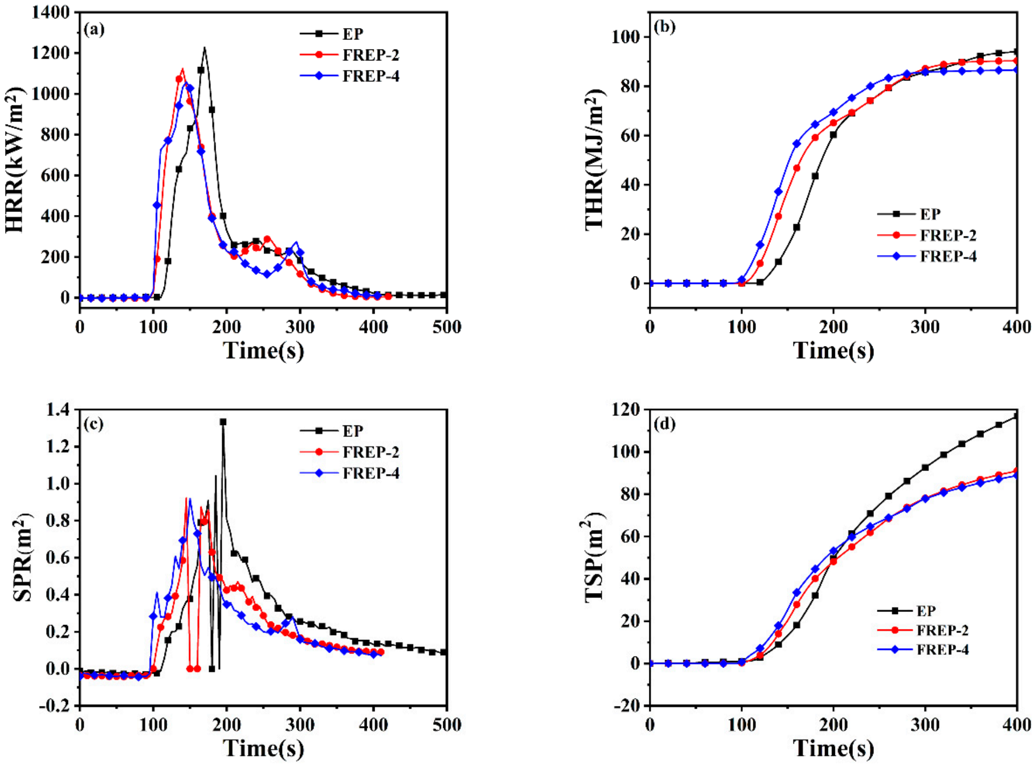

Figure S7 Variation of peak intensity of some typical flammable volatiles with time

(a) hydrocarbons, (b) aromatic compounds, (c)carbonyl compound, and (d) aliphatic ethers

8. Glass transition temperature (T_g)

T_g was measured on a Perkin-Elmer DSC 4000 (PE, USA) at a heating rate of 10 °C /min.

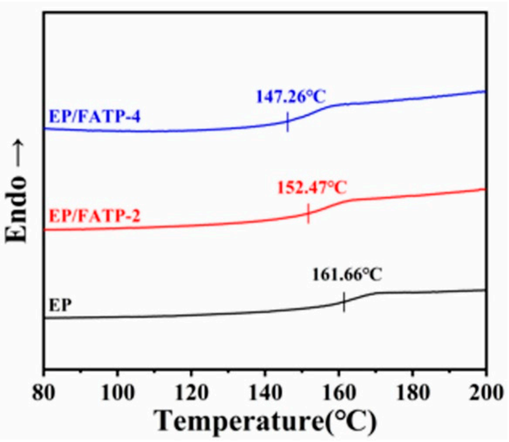

Figure S8 DSC curves of pure EP and FREPs

9. Dynamic mechanical analysis (DMA)

DMA was carried out on a Perkin Elmer DMA 8000 (PE, USA) using a three-point bending mode in temperature range of 30~250 ℃ at a heating rate of 10 ℃/min, the size of the sample is 40 mm×6 mm×3 mm.

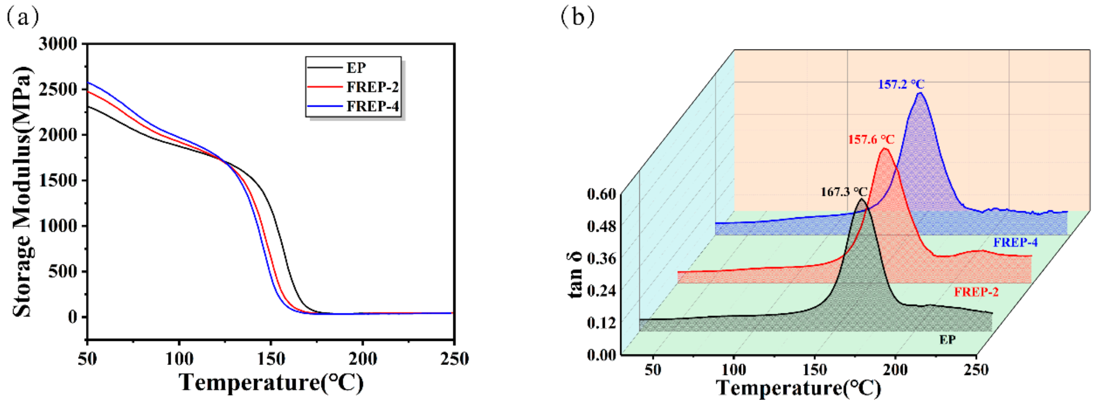

Figure S9 DMA curves of pure EP and FREPs
